# The activity of BcsZ of *Salmonella* Typhimurium and its role in *Salmonella*-plants interactions

**DOI:** 10.3389/fcimb.2022.967796

**Published:** 2022-08-23

**Authors:** Ilana S. Fratty, Dina Shachar, Marina Katsman, Sima Yaron

**Affiliations:** Faculty of Biotechnology and Food Engineering, Technion – Israel Institute of Technology, Haifa, Israel

**Keywords:** food safety, *salmonella enterica*, foodborne pathogens, plant-*salmonella* interactions, biofilm

## Abstract

*Salmonella enterica* is one of the most common human pathogens associated with fresh produce outbreaks. The present study suggests that expression of BcsZ, one of the proteins in the *bcs* complex, enhances the survival of *Salmonella* Typhimurium on parsley. BcsZ demonstrated glucanase activity with the substrates carboxymethylcellulose and crystalline cellulose, and was responsible for a major part of the *S*. Typhimurium CMCase activity. Moreover, there was constitutive expression of BcsZ, which was also manifested after exposure to plant polysaccharides and parsley-leaf extract. In an *in-planta* model, overexpression of BcsZ significantly improved the epiphytic and endophytic survival of *S*. Typhimurium on/in parsley leaves compared with the wild-type strain and *bcsZ* null mutant. Interestingly, necrotic lesions appeared on the parsley leaf after infiltration of *Salmonella* overexpressing BcsZ, while infiltration of the wild-type *S*. Typhimurium did not cause any visible symptoms. Infiltration of purified BcsZ enzyme, or its degradation products also caused symptoms on parsley leaves. We suggest that the BcsZ degradation products trigger the plant’s defense response, causing local necrotic symptoms. These results indicate that BcsZ plays an important role in the *Salmonella*-plant interactions, and imply that injured bacteria may take part in these interactions.

## Introduction

Non-typhoidal *Salmonellae* (NTS) serotypes cause foodborne illness worldwide, with increasing risk of severe diseases in the two extremes of age, and high mortality rates in the elderly (Solnik- Isaac et al., 2007; Zaidenstein et al., 2010; Weinberger et al., 2011; Alegbeleye et al., 2018). Since NTS serotypes are widespread in nature, and have the ability to adapt to various environmental conditions, they are found in many foods, including leafy greens, fruits and vegetables. Consequently, NTS have been rated as the leading cause of bacterial foodborne diseases and outbreaks associated with consumption of fresh produce (Fisher et al., 2015). Microbial contamination of produce may occur at any stage from farm to fork (European Food Safety Authority, 2017). Furthermore, studies have indicated that some serotypes like *S. enterica* serotype Typhimurium are capable of surviving in leafy greens until harvest and during processing (Brandl, 2006; Kisluk and Yaron, 2012; Szpinak et al., 2022). The inefficacy of disinfectants in removal or inactivation of attached or invaded *S*. Typhimurium (Shirron et al., 2009), and the absence of visual signs for contamination on plants (Lapidot et al., 2006), further contribute to the relative high number of outbreaks (Montano et al., 2020).

Irreversible adhesion of *S*. Typhimurium on the plants’ edible parts was demonstrated on lettuce, basil, parsley, peppers, tomatoes, and more (Lapidot and Yaron, 2009; Kroupitski et al., 2011; Shaw et al., 2011, Kisluk and Yaron, 2012). The attachment to the phyllosphere may be followed by either colonization and biofilm formation on the surface (Lapidot et al., 2006), entry into the host tissue (Kroupitski et al., 2011), or invasion into the plant cells (Schikora et al., 2008; Shirron and Yaron, 2011). Endophytic survival helps the bacteria to escape from the hostile and instable environment on the leaf surface, but forces the bacteria to cope with new challenges like the plant defense response.

The frequent cases of NTS outbreaks associated with fresh produce, together with experimental evidence strongly suggest that NTS acquired mechanisms for survival in plants (Martinez-Vaz et al., 2014). Despite extensive research about the attachment, invasion, and survival of *Salmonella* on plants, the mechanisms by which *Salmonella* survives, and especially the ways it interacts with the plant defense systems, have not been fully understood. Identification of strategies that enhance the survival of *S*. Typhimurium in plants can be obtained by comparing between *S*. Typhimurium and known phytopathogens. *S*. Typhimurium and phytopathogens share genes involved in host-pathogen interactions such as genes encoding aggregative fimbriae and curli, Type 3 secretion systems, secreted effector proteins, and polysaccharides producing enzymes, which commonly function in similar ways during colonization in the relevant host (Barak et al., 2005; Jeter and Matthysse, 2005; Barak et al., 2007; Shirron and Yaron, 2011). Key factors that play an essential role in the survival of phytopathogens in their host, but have not been fully addressed in human pathogens colonizing plants, are cell wall degrading enzymes (CWDEs). A major part of CWDEs demonstrate β-glucanase activity, meaning they hydrolyze polysaccharides made up of β-linked monosaccharides, like cellulose and xylan. Phytopathogens’ CWDEs take part in the bacteria-host interactions, because they help to overcome the barrier of the plant cell wall during entery and spread, increase the availability of nutrients and energy during colonization and survival, and improve the ability of the pathogens to translocate effector proteins and toxins to the host cells (Walton, 1994; Kubicek et al., 2014). *S*. Typhimurium produces at least one carboxymethylcellulase (CMCase), BcsZ (also known as CelC). This enzyme contains conserved motifs, which indicate its belonging to glycoside hydrolases family 8 (GH8) (Yoo et al., 2004). Moreover, BcsZ has a strong homology to CWDEs found in phytopathogens such as *Cellulomonas uda* (Romling and Galperin, 2015), *Erwinia chrysanthemi* (Solano et al., 2002), and *Agrobacterium tumefaciens* (Matthysse et al., 1995). Therefore, the main objective of this study was to study the role of BcsZ in the plant-*Salmonella* interactions.

## Results

### Glucanase activity of BcsZ

**Figure 1:**
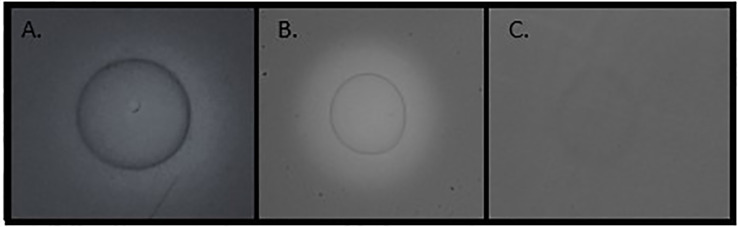
Degradation of carboxymethylcellulose (CMC) on CMC agar plates by bacterial lysate. Overnight grown cultures were sonicated and centrifuged, and the supernatant was spotted on CMC agar plates. The plates were stained with 0.1% Congo red and washed with 1M NaCl. Bright halos indicate CMC degradation. **(A)** Bacterial lysate of *S*. Typhimurium (w.t.), a light halo is observed, circulating the bacterial lysate. **(B)** Bacterial lysate of *S*. Typhimurium pBcsZ (overexpressing BcsZ), a bright and strong halo is observed **(C)** Bacterial lysate of *S*. Typhimurium ΔBcsZ (lacking the ability to produce BcsZ), no halo is observed.

The first objective of the study was to investigate if *S*. Typhimurium expresses enzymes with glucanase activity. Carboxymethylcellulose (CMC), which has extensively been used to study glucanase activity, served as a substrate in this assay. CMC is a soluble cellulose derivative with carboxymethyl groups bound to some of the hydroxyl groups of its glucopyranose monomers. CMC degradation activity of *S*. Typhimurium colonies was investigated on CMC agar plates using the Congo red staining method. Clear halos are expected to appear on the CMC ager plates after Congo red staining, in the presence of an extracellular CMCase activity (Teather and Wood, 1982; Yoo et al., 2004; Xia et al., 2016). When the *S*. Typhimurium wild type (w.t.) strain was grown on CMC plates and stained with Congo red, no CMC degradation was observed. In fact, the bacteria were colored, with dark red color and red halos around them, probably due to bacterial cellulose synthesis (**Figure S1A**). Considering BcsZ’s periplasmic location (Ahmad et al., 2016), we repeated the experiment using bacterial lysates (**Figure 1**). The w.t. lysate had a slight bright halo, indicating a weak CMCase activity (**Figure 1A**). To further determine if the CMCase activity is related to BcsZ, the experiment was repeated with lysate of the ΔBcsZ mutant. ΔBcsZ had no halo zone around the bacterial lysate (**Figure 1C**), revealing that CMCase activity of *S*. Typhimurium is dominating by BcsZ. The CMCase activity of bacteria that overexpress BcsZ was investigated, to study the potential activity under conditions that induce the expression of this protein, or in strains that naturally produce higher amounts of BcsZ. *S*. Typhimurium pBcsZ, had a strong halo zone, more notable than that of the w.t. (**Figure 1B**). *S.* Typhimurium w.t. strain harboring the *pCA114* plasmid without gene insertion demonstrated the same activity as the w.t strain, indicating high CMCase activity in the BcsZ overexpressing strain. A similar result was obtained with the complemented mutant, in which ΔBcsZ was transformed with pBcsZ plasmid (**Figure S1B**).

**Figure 2:**
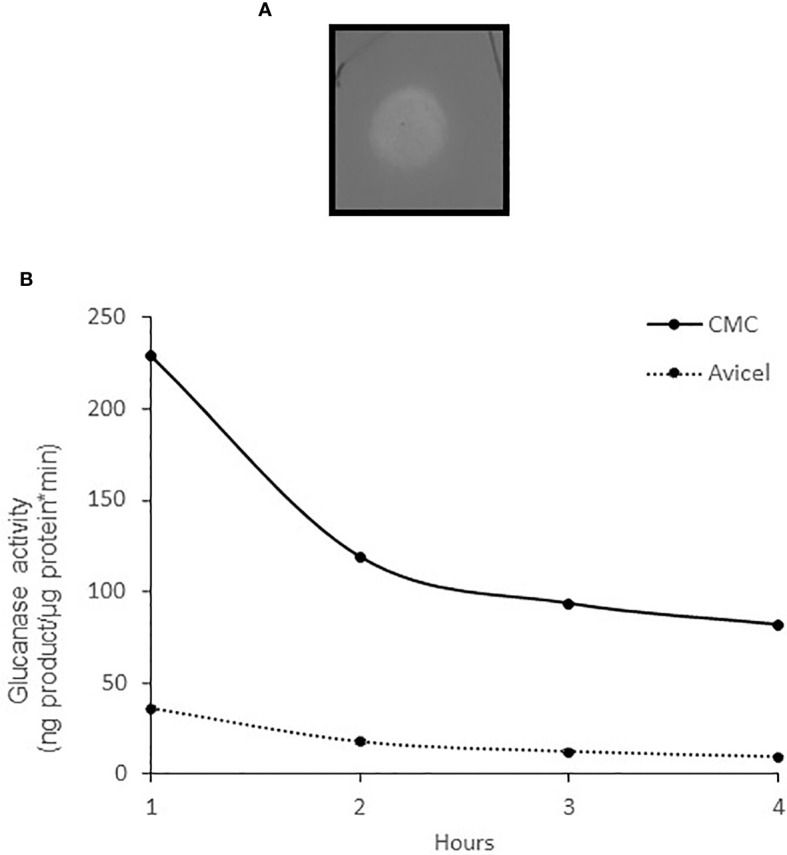
The activity of the purified enzyme r-BcsZ. **(A)** r-BcsZ enzyme was expressed in *E. coli*, purified, and spotted on CMC agar plates. The plates were stained with 0.1% Congo red and washed with 1M NaCl. A bright halo is observed, indicating CMC degradation. **(B)** Glucanase specific activity of r-BcsZ measured with BCA assay using CMC and Avicel as substrates.

To measure the enzyme activity quantitatively, we amplified the *bcsZ* gene of *S*. Typhimurium by PCR, cloned the gene in an expression vector, expressed the recombinant BcsZ protein (r-BcsZ) in *E. coli*, and purified it. The activity of the recombinant enzyme was measured using the BCA assay (Kongruang et al., 2004) with 1% CMC or 1% crystalline cellulose (Avicel), and on CMC agar plates. The CMC’s amorphous structure makes it a suitable substrate for endoglucanases, whereas crystalline cellulose such as Avicel is degraded primarily by exoglucanases. The enzyme caused a clear yellow zone on CMC agar plates (**Figure 2A**), and showed a stronger activity on CMC compared with Avicel (**Figures 2B**).

### Activity of the bcs operons in the presence of polysaccharides and extract of parsley leaves

**Figure 3:**
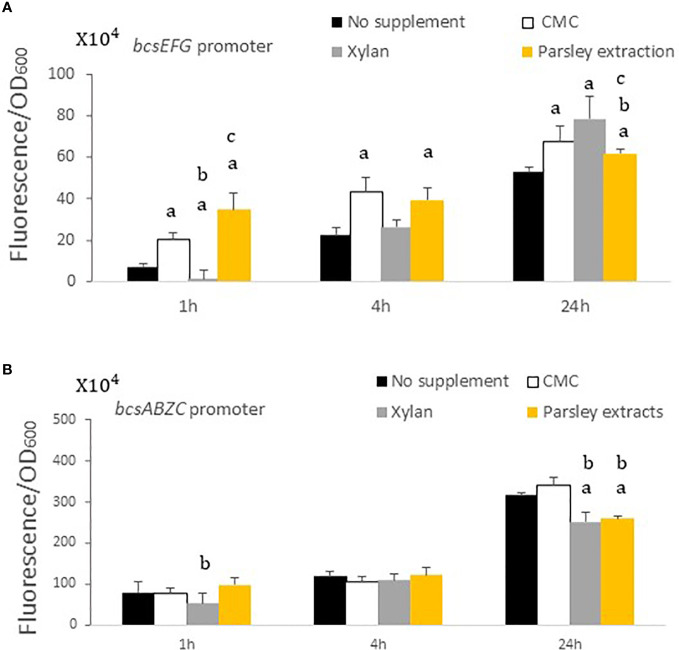
The effects of plant components on the *bcsEFG* and *bcsABZC* expression. *S*. Typhimurium containing a plasmid with either *bcsEFG* or *bcsABZC* promoter fused upstream to a *gfp* gene was grown in minimal medium in the presence of CMC, xylan or parsley extracts. The fluorescence was measured 1, 4 and 24 hours post polysaccharides addition. **(A)** Normalized fluorescence measurements of GFP with *bcsEFG* as promoter in the presence of CMC, xylan and parsley extracts. **(B)** Normalized fluorescence measurements of GFP with *bcsABZC* as promoter in the presence of CMC, xylan and parsley extracts. Mean values of four experiments, each one was conducted in triplicates, are presented. Error bars represent standard deviations. Statistical calculations are represented as a, b and c when “a” defines the significance difference between no supplement to other treatments at the same time point, “b” defines CMC to other treatments and “c” defines the difference between xylan and parsley extracts. *P* ≤ 0.05.

The *bcsABZC* and *bcsEFG* operons are located in the bacterial cellulose synthesis site, in close positions with opposite orientations (Solano et al., 2002). We used the GFP as a reporting protein to study the activity of both promoters in the presence of plant carbohydrates. The *gfp* gene was fused to each promoter, cloned in the pCS21 plasmid, and the resultant plasmids were transformed to w.t. *S*. Typhimurium. The bacteria harboring pCS21/*pbcsABZC-gfp* and pCS21/*pbcsEFG-gfp* were grown in a minimal medium containing either CMC, xylan, or extract of parsley leaf, and the normalized GFP fluorescence was measured. The basal GFP fluorescence level under the *bcsABZC* promoter was significantly higher compared with the expression levels under the *bcsEFG* promoter (**Figure 3**). Fluorescence of GFP fused to the *bcsEFG* promoter increased in the presence of CMC and parsley extract after 1 and 4 hours, and in the presence of all three supplemented substances after 24 hours. Whereas GFP expression under the *bcsABZC* promoter did not change in the presence of the polysaccharides nor the leaves extract after 1 and 4 hours, and decreased significantly with xylan and parsley extract after 24 hours. Still, fluorescence of GFP under the *bcsABZC* promoter was much higher, with and without the supplemented substances (**Figure 3**), (*P ≤* 0.05, based on T-Test statistical analysis).

### Role of BcsZ in survival of *S*. Typhimurium on and in parsley leaves

To examine the role of BcsZ in bacterial survival on plants, parsley plants were irrigated with water containing *S*. Typhimurium w.t., pBcsZ, and ΔBcsZ strains. Bacterial survival on leaves was tested 4 hours (time zero) and 7 days post irrigation (dpi), and bacterial reduction was calculated. No significant differences were observed in initial numbers of all three strains (**Table 1**), indicating that BcsZ does not affect the initial adhesion to the leaf. After 7 days, the bacterial counts of all strains decreased, meaning that none of them grew on the leaves. However, the BcsZ overexpressing strain exhibited the highest counts after 7 days (*P*<0.001 compared with both strains), indicating that this strain adapted to leaf conditions better than the others. Leaves irrigated with ΔBcsZ had the lowest bacterial survival (*P*=0.0618, compared with the w.t.) (**Table 1**).

**Table 1:** Survival of *S*. Typhimurium on parsley leaves.

	4 hours post irrigation	7 days post irrigation	Change after 7 days
Strain	Log (CFU/g) ± SD^a^		ΔLog (CFU/g) ± SD^a^
w.t.	6.3 ± 0.2	2.8 ± 0.3	3.5 ± 0.2
pBcsZ	6.1 ± 0.3	3.9 ± 0.2	2.2 ± 0.5*
ΔBcsZ	6.5 ± 0.3	2.4 ± 0.4	4.1 ± 0.7**

a Numbers represent means of 6 repeats, with standard deviations. Bacterial reduction addresses the delta of log S. Typhimurium counts between 4 hours and 7 dpi.

*A significant difference was observed between pBcsZ and both, w.t. and ΔBcsZ P < 0.001.

**A significant difference was observed between ΔBcsZ and w.t. P < 0.1.

**Figure 4:**
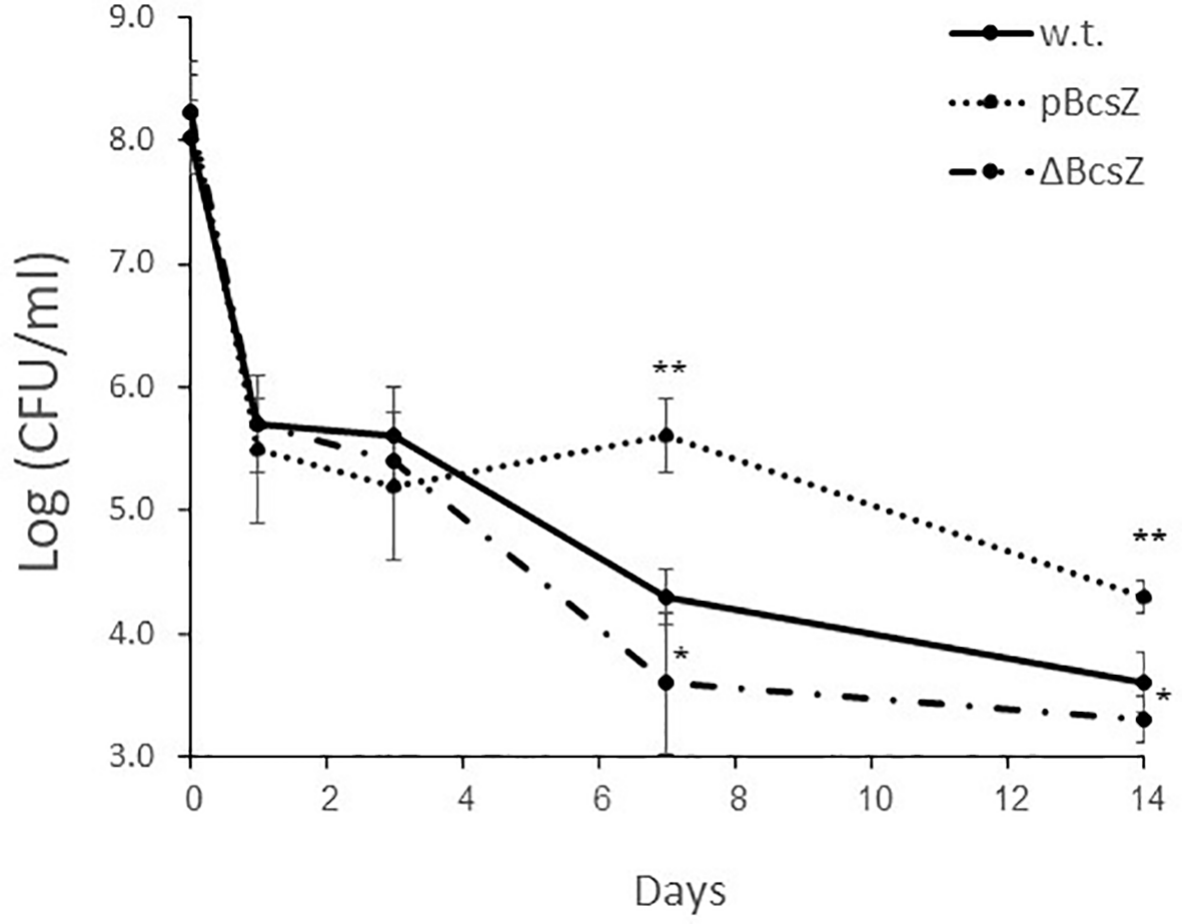
Endophytic survival of *S*. Typhimurium on parsley leaves. Parsley leaves were infiltrated with 8 ± 0.5 Log (CFU/ml) of bacterial solution containing w.t., pBcsZ and ΔBcsZ. Leaves were collected 1, 3, 7- and 14-days post infiltration, homogenized, diluted and plated for enumeration of viable *Salmonella*. Mean values of three experiments, each one was conducted in duplicates, are presented, and were statistically analyzed using Fisher’s protected least significant difference test (**P< 0.01, *P < 0.05). Error bars represent standard deviations.

The effect of BcsZ on the bacterial endophytic survival was in line with the epiphytic survival. All investigated strains survived in similar levels in the first days, and demonstrated more than 2 log reduction. However, the survival rates of pBcsZ, examined 7 and 14 dpi, were significantly higher comparing with the w.t. or ΔBcsZ strains (*P*<0.01), while bacterial counts of ΔBcsZ were significantly lower also compared with the w.t. (*P*<0.05) (**Figure 4**).

### Plant response to BcsZ

**Figure 5:**
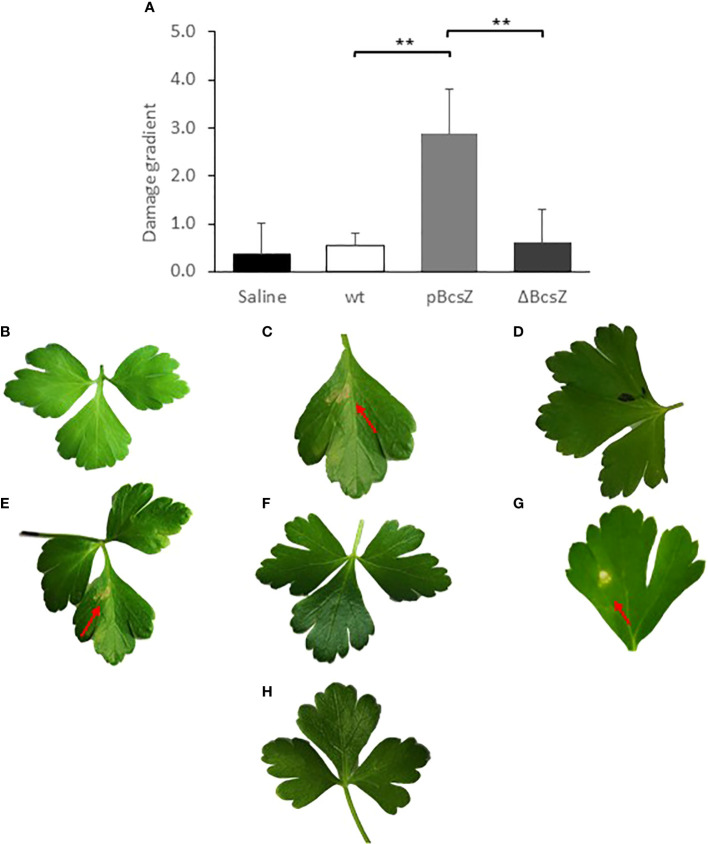
Necrotic damage on parsley leaves infiltrated with different *Salmonella* strains and solutions. **(A)** Summary of the necrotic damage intensity 7 days post infiltration. The damage in each leaf was scored according to a damage gradient described in **Figure S2**, and the mean scores of all leaves were calculated. The experiment was conducted in 3 repeats and statistical calculations were performed using Kruskal-Wallis test. **P < 0.001. Error bars represent standard deviations. B-H. Images of representative leaves. **(B)** Parsley leaves 7 days after bacterial infiltration of w.t., no leaf damage was observed. **(C)** Parsley leaves 7 days after pBcsZ infiltration, necrotic damage on leaves was observed. **(D)** Parsley leaves 7 days after bacterial infiltration of ΔBcsZ, no damage was observed. **(E)** Parsley leaves infiltrated with the purified r-BcsZ, necrotic leaf damage was observed. **(F)** Parsley leaves Infiltrated with buffer solution as control. **(G)** Parsley leaves infiltrated with degradation products of BcsZ, necrotic leaf damage was observed. **(H)** Infiltration of the denaturized enzyme r-BcsZ, no damage was observed. Red arrows point to the necrotic spots formed in the parsley leaf.

When we infiltrated the leaves with all strains, we did not observe noteworthy differences in plants growth between the control group and plants infiltrated with the bacteria. However, we noticed that, symptoms of local necrotic lesions started to appear in the pBcsZ-inoculated leaves 4 dpi, while leaves infected with the w.t. strain or ΔBcsZ looked healthy with no or minor signs of necrosis or damage even after 7 days or more. Images of representative leaves 7 dpi are shown (**Figures 5B-D**). Symptoms were scored according to their severity (**Figure S2**), and as can be seen in **Figures 5A**, the BcsZ overexpressing bacteria caused significant necrosis damages compared with the w.t. and the knockout mutant (*P <*0.001). To test if BscZ is responsible for the damage, we infiltrated the purified recombinant protein to the parsley leaves. Indeed, r-BcsZ caused local necrotic lesions 7 dpi (**Figure 5E**). As a control we infiltrated the buffer solution (excluding r-BcsZ) into parsley leaves and did not observe any necrotic spot (**Figure 5F**).

### The BcsZ degradation products induce the plant response

It was not clear which factor is responsible for triggering the plant response: the infiltrated proteins, their activity against the cell wall, or their degradation products. To address this question, r-BcsZ was mixed with 1% CMC and incubated to produce degradation products. Then, the solutions were separated by centrifugal concentrator to obtain two phases, the first phase contained substances larger than 10kD (including the enzymes r-BcsZ and undegraded polysaccharides), and the second phase contained the small molecules (including the degraded saccharides). The presence and absence of active r-BcsZ in both phases was confirmed on CMC agar plates. The phase with the larger molecules was then heated in order to inactivate the enzymes (as was confirmed on CMC agar plates stained with Congo red), and both phases were infiltrated into parsley leaves. Only the leaves infiltrated with the phase containing the CMC degradation products developed necrotic lesions in parsley leaves (**Figures 5G, H**).

## Discussion

Reports of food borne diseases caused by consumption of contaminated fresh produce, and studies of *Salmonella*-plant interactions suggest that *S*. *enterica* serotypes are capable of persisting on/in leaves, roots, fruits and seeds. They also support the hypothesis that *Salmonella* actively utilizes diverse strategies to adapt to the harsh environment in the plants in order to survive (Carstens et al., 2019, Lapidot et al., 2006; Kroupitski et al., 2009; Kisluk and Yaron, 2012; Kisluk et al., 2013; Tan et al., 2016; Chalupowicz et al., 2018). Indeed, an array of bacterial factors that impact the ability of *Salmonella* to attach, invade, and colonize plants has been described (Yaron and Romling, 2014). Yet, there has been no study on the potential role of glucanases in the *Salmonella*-plant interactions, even though similar enzymes have a crucial role in the pathogenicity of phytopathogens (Gough et al., 1988; Roberts et al., 1988). This may relate to a previous assumption saying that members of the Enterobacteriaceae family lack CWDEs (Teplitski et al., 2012). In this study we demonstrated that BcsZ of *Salmonella* has high activity compared with other bacterial glucanases (Jha et al., 2007). Moreover, we demonstrated that BcsZ affects the survival of *Salmonella* in plants in long-term. Detecting active glucanases in enteric pathogens has a great importance, because it points to potential mechanisms of survival of the bacteria in the plants.

Phytopathogens and symbiotic bacteria produce CWDEs (Gough et al., 1988; Roberts et al., 1988; Gibson et al., 2011), which facilitate their survival by various mechanisms (Gough et al., 1988; Roberts et al., 1988; Xia et al., 2016). In most cases, microorganisms that have evolved to degrade and utilize the plant cell wall, produce and secret an array of CWDEs with different activities that together, allow an efficient degradation of the diverse polymers of the cell wall (Walton, 1994; Bayer et al., 1995; Souza et al., 2017). According to the CAZy Database (Haft et al., 2003), *S*. Typhimurium has 95 potential carbohydrate-active enzymes (http://www.cazy.org), but there are only few putative glucanases, and BcsZ is the only confirmed glycoside hydrolase of family 8 that is capable of degrading 1-4-β-glucosidic bonds (Yoo et al., 2004; Mazur and Zimmer, 2011; Ahmad et al., 2016). The present study confirms that CMC and Avicel can serve as substrates of BcsZ. Furthermore, the basal expression of BcsZ was high, also in presence of polysaccharides or parsley extracts, consistent with other studies (Ahmad et al., 2016). Lysates of *S*. Typhimurium ΔBcsZ cells lost its glucanase activity, hence, it can be concluded that BcsZ is responsible for the major part of the *S*. Typhimurium CMCase activity *in-vitro*. The absence of other dominant glucanases supports the hypothesis that *S*. Typhimurium is not a classical phytopathogen (Lapidot and Yaron, 2009). Moreover, the evidence that BcsZ is not secreted into the medium, and is not induced by polysaccharides indicates that *S*. Typhimurium does not use BcsZ for obtaining a significant amount of nutrients in the plant environment. CWDEs of some microorganisms facilitate the adhesion of bacteria to the plant cells by attaching to the cell wall (Bayer et al., 1995). We did not observe changes in the ability of *Salmonella* to attach to the parsley cells in the absence of BcsZ, meaning that BcsZ does not have a dominant role in the initial attachment. On the other hand, the results of this study demonstrate that BcsZ contributes to the long-term survival of *S*. Typhimurium on the plants.

BcsZ is a component of the cellulose producing machinery, and its role in biosynthesis of the *Salmonella*’s cellulose was proposed to be in the cleavage of the translocating glucan chains (Mazur and Zimmer, 2011). Enzymes of the cellulose production machinery are necessary for *Salmonella* survival in different hosts, including plants, mainly due to the protection effects of the biofilm, and its importance in attachment (Barak et al., 2007; Lapidot and Yaron, 2009; Shaw et al., 2011; Martinez-Vaz et al., 2014; Romling and Galperin, 2015). Based on that, it could have been suggested that BcsZ contributes to the *Salmonella* survival on the plants through biofilm production. However, biofilm production does not fully explain the results of this study, because when overexpressed, BcsZ negatively regulates cellulose biosynthesis by downregulation of the cellulose synthesis enzymes (Ahmad et al., 2016). It means, that less biofilm is produced in the BcsZ overexpressing strain that also better survives in and on the leaves. These results point to the conclusion that BcsZ functions in both, hydrolytic activity and cellulose production, and both functions have a role in the survival of *S*. Typhimurium on plants. The multifunctional activity of enzymes from the cellulose biosynthesis machinery in colonization of plants was demonstrated with other enzymes of phytopathogens and symbiotic bacteria. For example, BcsZ of *Rhizobium leguminosarum* takes part in cellulose synthesis, but also degrades cellulose in the hair wall at the root tips in order to initiate colonization (Robledo et al., 2012).

The other role of BcsZ in the *Salmonella*-parsley interactions is revealed by the observation that overexpression of BcsZ induced the plant’s defense response. It has already been shown that degradation of plant polysaccharides triggers the plant immune response (Jha et al., 2007; Hematy et al., 2009; Bellincampi et al., 2014). Moreover, cellulases and xylanses of *X*. *oryzae* pv. *Oryzae* caused hypersensitive response (HR) symptoms (Jha et al., 2007). Such observations correlate with our observations, in which r-BcsZ caused necrotic areas on the leaves. Induction of HR may occur by several triggers. Conserved sequences of the enzymes may be recognized as pathogen-associated molecular patterns (PUMPs) (Bellincampi et al., 2014). Loss of cell wall integrity is normally accompanied by changes in plant cell shape and size, which can also elicit the plant defense system (Souza et al., 2017). In addition, release of oligosaccharides produces damage signaling in plants, that triggers the production of plant defense hormones (Hematy et al., 2009; Bellincampi et al., 2014; Xia et al., 2016). Arabidopsis, for example, activates its immune system when perceiving small oligomers of cellulose and cellobiose (Souza et al., 2017). As seen in **Figure 5**, infiltration of the degradation products of BcsZ resulted with the appearance of clear necrotic spots on the parsley leaves. According to these results, degraded oligosaccharides in parsley are most likely sensed by the plant immune system and cause HR symptoms, reveals that BcsZ triggers the plant immune response indirectly by releasing degradation products.

A further question arises from these experiments, how BcsZ is capable of damaging parsley leaves. One possible answer is that when BcsZ is overexpressed it is secreted from the cell. A more suitable suggestion is that BcsZ is poured out from the injured *Salmonella* cells to the extracellular space. As we have reported in our previous studies (Lapidot and Yaron, 2009; Kisluk and Yaron, 2012), and enforced in this study, the number of bacterial cells considerably decreases after being inoculated on the plants (**Figure 4**). In other words, more than 99% of the bacteria do not survive, and therefore BcsZ is possibly released from the periplasm of the injured or dead bacteria, and encounters the adjacent plant cell wall, or the cellulose of the bacterial biofilm, if had been formed. When BcsZ degrades the polymers, it can provide a low amount of nutrients and enables better bacterial penetration and spread, which may enhance the survival of the small portion of live cells. Moreover, not only infiltration of live and purified r-BcsZ caused the HR symptoms, but also the infiltration of bacterial lysate expressing BcsZ. Lysates of *S.* Senftenberg, for example, were also shown to induce chlorosis in *A. thaliana* (Berger et al., 2011), however the trigger was not identified, and we suggest that glucanase had a role in this observation, as they do in phytopathogens such as *X*. *oryzae* pv. *Oryzae* (Jha et al., 2007).

Here we propose that the main role of *Salmonella*’s BcsZ is in the process of cellulose synthesis for biofilm formation, but under specific circumstances, its ability to function as a glucanase and its constitutive expression may affect the interactions between *Salmonella* and the host plant. In this study we constructed *S*. Typhimurium that overexpresses BcsZ, but previous observations have shown that *S*. Agona, Montevideo and Senftenberg isolates are more effective at producing biofilms (Vestby et al., 2009). These biofilm producers naturally produce high amount of cellulose (Solano et al., 2002), express higher amounts of the bacterial cellulose synthesis proteins, and their lysates have much higher CMCase activity, compared with the *S*. Typhimurium strain used in this study (**Figure S3**). Hence, these strains have the potential to degrade plant polysaccharides more effectively. Interestingly, *Salmonella* isolates from fresh produce are moderate or strong biofilm producers (Amrutha et al., 2017). In other words, it is possible to find isolates that have higher expression of BcsZ, and these strains will be found more frequently in plants than in mammalian hosts (Martinez-Vaz et al., 2014).

## Conclusion

A new mechanism of plant-*Salmonella* interactions is suggested, in which the glucanase BcsZ of *S*. Typhimurium affects bacterial survival on and in plants and is responsible for triggering the plant immune response by causing HR symptoms *via* partial degradation of polysaccharides. There is an argument whether plants are true alternative hosts for *Salmonella*, or whether they are just serving as an environment where *Salmonella* cells persist as part of the natural life cycle, until they move into a new host. We propose that *Salmonella*, which has incredible abilities to adapt to environmental changes, also managed to adapt to the plant’s matrices, by relying on existing systems and enzymes important for survival in animal hosts or in the environment. The significance of the present study is high since we suggest, for the first time, the possibility that injured or damaged cells also contribute to the *Salmonella*-plant interactions. A further study is conducted to investigate if the substrates of the released glucanases are polymers of the plant cell wall, or the bacterial biofilm matrix. Moreover, the role of other putative CWDEs of *Salmonella* should be investigated.

## Experimental procedures

### Bacterial strains and growth conditions

The major bacterial strain used in this study was *Salmonella enterica* serotype Typhimurium ATCC 14028 (*S*. Typhimurium). This strain was extensively studied with regards to attachment and survival in fruit, vegetables and leafy greens (Crook et al., 2003). For protein expression and purification, we used the *E. coli* K12 BL21 strain (Ben-Barak et al., 2006). Unless otherwise described, bacteria were grown on liquid Luria-Bertani (LB) medium. When required, ampicillin (100 µg/ml), chloramphenicol (30 µg/ml) or kanamycin (30 µg/ml) were supplemented to the growth media. For overexpression experiments of BcsZ in *S*. Typhimurium, L-arabinose (20 µg/ml) was added.

### Generation of knockout and overexpressing *S*. Typhimurium strains

The expression vector pCA114 (AMP^r^) was used to construct an overexpressing BcsZ *Salmonella* strain under the control of *araBAD* promoter. All DNA techniques were carried out as described earlier (Ben-Barak et al., 2006; Hartog et al., 2008). Briefly, the *bcsZ* gene was amplified from the genome of *S.* Typhimurium 14028 by PCR using the oligonucleotides listed in Table S1. PCR products were digested using the restriction enzymes EcoR1 and Xba1 (New England Biolabs, USA), and ligated to the pre-digested pCA114 plasmid. Insertion was confirmed by PCR and subsequently by sequencing, and the plasmid was then transformed to *S*. Typhimurium 14028 and to its BcsZ null mutant by electroporation. Colonies carry the pBcsZ plasmid were grown in LB supplemented with 0.2% L-arabinose (Merck, Germany) for expression. Control was *S.* Typhimurium w.t. strain harboring the *pCA114* plasmid without gene insertion.

The site-specific deletion of *bcsZ* was constructed essentially according to the λ red-mediated gene replacement protocol (Datsenko and Wanner, 2000) using the primer pairs described in Table S1. Briefly, electro-competent *S.* Typhimurium cells were transformed with pKD46 plasmid to make the *S.* Typhimurium (+pKD46) strain. pKD3 plasmid, containing the *cat* gene was amplified by PCR using primers described in Table S1 to obtain a linear fragment of the cassette with FRT (FLP Recognition Target) sequence, flanked by ~50 bp upstream and downstream of the target *bcsZ* gene. PCR products were verified on an agarose gel and purified from the gel using gel purification kit (QIAquick Gel Extraction Kit, Qiagen). Linear fragments (~50 ng) were transformed into competent *S.* Typhimurium (+pKD46) by electroporation. Transformants were incubated in LB broth for 1.5 h at 37°C, and grown overnight on LB agar plates with chloramphenicol (10 µg/ml) at 37°C, for selection. The correctness of the mutation was confirmed by colony-PCR using primers pairs aimed to confirm that the *cat* gene was inserted and the target gene was deleted. Additionally, sequencing was performed, to verify that the *cat* gene has replaced the target gene. This procedure allowed for the construction of a single Δ*bcsZ* knockout *S*. Typhimurium strain.

### Cloning, expression and purification of recombinant BcsZ in *E. coli*

The *bcsZ* gene (excluding the leader sequence and fused to His-tag on the amine side) was amplified from the genome of *S.* Typhimurium 14028 using PCR with the primers described in Table S1. After amplifying the gene, the PCR products were digested with BamH1 and Nco1 and inserted into pre-cut pET-9d plasmid using the ligase enzyme. The plasmids were then transformed into competent *E. coli* BL21 cells by electroporation for inducible overexpression. Insertion was confirmed by PCR and subsequently by sequencing. Purification of the recombinant protein was carried out according to a previously published procedure (Wang et al., 2005), with modifications. Briefly, a bacterial colony was grown overnight at 37°C with shaking. The overnight culture was diluted 1:100 in 800ml LB broth and incubated at 37°C. When the OD600 was ~0.6, 1mM IPTG was added to the culture and growth was continued for 16h. The cells were harvested by centrifugation at 4000g for 20 min at 4°C, followed by resuspension in 20mM NaH_2_PO_4_ (200ml) and centrifugation. The cells pellet was suspended in 35ml lysis buffer and homogenized by an EmulsiFlex-C3 (Avestin, Canada). Following centrifugation of the cell lysates at 10,000g for 30min at 4°C, the supernatant was gently mixed with 1ml of pre-washed Ni-NTA resin beads (Qiagen) for 90min at 4°C, to allow binding of the proteins to the nickel-NTA beads. The mixture was applied to a Bio-Rad gravity flow column and the beads were washed with 20ml of wash buffer A, containing 20mM imidazole and with 40ml of wash buffer B, containing 50mM imidazole. Proteins were eluted with 15ml elution buffer, containing 250mM imidazole. Protein purity was assessed by SDS-PAGE after Coomassie blue staining, and the final concentration was estimated using the Bradford protein assay using Bio-Rad reagent (Bio-Rad laboratories, USA). Finally, the sequence of the purified protein, termed r-BcsZ, was verified using mass spectrometry at the Smoler protein research center at the Technion as described (Lehmann et al., 2016), and was found to match the BcsZ protein of *S*. Typhimurium.

### Degradation of CMC by BcsZ

Each bacterial strain (8 ml) was grown overnight in LB with shaking. Following centrifugation, bacteria were resuspended in 50mM Tris HCl and sonicated by an ultrasonic liquid (9.9s followed by 5.5s of resting processor) for 3 min (Sonics Vibra cell 505, USA). After centrifugation of the bacterial lysates, the upper supernatant was further examined. Each bacterial lysate (20µl) was spotted on 0.2% CMC (low viscosity, Sigma, USA) agar plates and incubated at 37°C overnight. The incubated plates were stained with 0.1% Congo red for 30 minutes, rinsed with water and then washed twice with 1 M NaCl. Cellulose degradation is shown as a white halo against dark background (Teather and Wood, 1982; Yoo et al., 2004; Xia et al., 2016). For examination of cellulase activity by r-BcsZ, the purified protein (10μg) was spotted on 1% CMC agar plates and treated similarly to the bacterial lysates.

### Study the kinetics of degradation using the BCA assay

Purified r-BcsZ was incubated with 1% CMC or 1% Avicel for one hour at 40°C (Yoo et al., 2004). Degradation of the substrates was measured by the Reducing sugar assay using bicinchoninate acid (BCA, Tokyo Chemical Industry) as described (Zhang and Lynd, 2005), and results were normalized to the control containing the substrates without enzymes. Purified glucose was used for standard curves.

### Detection of promoter activity

The promoter’s activity was measured using the green fluorescent protein (GFP) as a reporter protein. The *bcsABZC* and *bcsEFG* promoters were amplified from the genome of *S*. Typhimurium, fused to the *gfp* gene (*gfpmut3*), and cloned in the low-copy vector pCS21 as described (Ben-Barak et al., 2006). These reporting plasmids were generously obtained from Dr Keren Scher, and were based on the 5’ Race assay (Invitrogen), and an analysis through the site Neural Network Promoter Prediction (NNPP). Primers used for cloning are listed in the supplementary table (Table S1). The cloned plasmids (*pCS21/pbcsABZC-gfp* and *pCS21/pbcsEFG-gfp*) were transformed to w.t. *S*. Typhimurium cells. Overnight cultures of *S*. Typhimurium harboring each of the GFP plasmid were harvested by centrifugation (4000 X g for 10 min at 4°C) and diluted 1:10 in Minimal Medium (0.2% NaNO_3_, 0.1% K_2_HPO_4_, 0.05% MgSO_4_, 0.05% KCl, 0.5% Peptone) supplemented with 30µg/ml kanamycin. Each sample was supplemented with 0.5% CMC, 0.5% xylan or parsley extract (20gr parsley leaves mixed with 200 ml dH_2_O and homogenized in a stomacher twice for 90 seconds each time), and incubated for 24 hours. Control samples were not supplemented with plant components. Fluorescence (filters F485, F535, CW lamp energy 10,000) and absorbance (OD) (600nm P600 filter) measurements were performed using the WallacVictor2 multi-well fluorimeter. Experiments were repeated 4 times in triplicates. The fluorescence intensity (arbitrary units UA) and OD measurements were normalized and calculated as described (Hartog et al, 2008).

### Plant growth

Parsley plants (*Petroselinum crispum* var. *neapolitanum*) were grown in a greenhouse. Parsley seeds were disseminated in each planter containing 12 liters (for irrigation procedure) or 2.5 liter (for infiltration procedure) of commercial nonsterile potting soil (Avital 11; Tuff Marom Golan). The size of the planters used in this study was 40/20/19cm (length/width/height) for the irrigation experiments or round pots (17cm diameter and 12-16cm height) for infiltration experiments. The plants were automatically watered with tap water using drip irrigation. At the age of 10 to 12 weeks the parsley plants (at least 10 in each planter) were able to provide enough mass for sampling as described in other studies in this field (Kisluk and Yaron, 2012).

### Contamination of parsley plants by irrigation with contaminated water

Plants (at the age of 10-12 weeks) were manually sprayed with a hand sprayer with 150 ml of saline/bacterial solution. The bacterial solution contained 10^8^ CFU/ml of different bacterial strains. For control, planters were irrigated with sterile saline. For safety reasons, the irrigation of each planter was conducted inside a glove box, from which the plant was removed 1 h after irrigation. Parsley leaves were harvested 4 hours and 7 days post irrigation. The leaves were then aseptically weighted (10gr), and mushed in a stomacher for enumeration of the bacteria by plate counting as described (Kisluk and Yaron, 2012).

### Contamination of parsley plants by infiltration of bacterial cultures or proteins

For analysis of endophytic survival, suspensions of 10^8^ CFU/ml *Salmonella* in saline were infiltrated into parsley leaves. Infiltration of bacterial suspensions were carried out by infiltrating 25 µl to the abaxial surface of the leaf (20 leaves in each plant, 3 plants in each experiment) as described before (Shirron and Yaron, 2011). Parsley leaves were harvested 1, 7, and 14 dpi. Leaves were aseptically collected, weighted, added to peptone buffer (1:10 dilution), and homogenized in a stomacher. The supernatant was plated for CFU enumeration. All experiments were conducted at least 3 times in duplicates. Bacteria lysates of overnight grown cultures were produced as described above in the CMCase assay, and 25 µl were used for the infiltration.

For infiltration of purified proteins, we infiltrated various concentrations of purified r-BcsZ (0.1µg to 100 µg) to the abaxial side of the leaf similarly to bacterial inoculation. For infiltration of denaturized r-BcsZ and its degradation products, the enzyme was mixed with 1% CMC for 1h, then the two fractions were separated using a 10,000 MWCO Vivaspin^®^ centrifugal concentrator (Vivaproducts, MA, USA). As a negative control, 1% CMC was treated without enzymes. The concentrated recombinant enzymes from the concentrator tubes were treated at 90°C in a water bath for 15min prior to the infiltration. The solutions that did not contain the recombinant enzymes were infiltrated into parsley leaves without further treatments. For verification, CMCase activity of each fraction was analyzed on CMC agar plates as described above.

### Statistical analysis

Unless mentioned specifically, all experiments were conducted in three repeats. Sampling of each experimental repeat was conducted in duplicates. Results of survival of *Salmonella* on/in leaves were statistically analyzed using Fishers’s protected least significant difference test, and results of the leaves damage were statistically analyzed with Kruskal–Wallis test. For statistics calculations in the GFP expression experiments we used T-Test. All statistical analyses were performed in MATLAB 2019b (The Mathworks, Natick, USA).

## Data availability statement

The original contributions presented in the study are included in the article/**Supplementary Material**. Further inquiries can be directed to the corresponding author.

## Author contributions

All authors contributed to the study design. IF conducted experiments, designed the figures, wrote the original draft and performed the statistical analysis. IF, DS and MK were involved in the cloning of bacterial strains. SY conceived the study, and was involved in interpretation of the data and in critical revision of the manuscript. The corresponding author confirms that she had full access to all the data in the study. All authors contributed to the article and approved the submitted version.

## Funding

This work was supported by the Israel Science Foundation (ISF) grant No 914/11, and by the Technion Russel Berrie Nanotechnology Institute (RBNI).

## Acknowledgments

We would like to thank Dr. Keren Scher for the *pCS21/gfp* plasmids, and Shira Reznik Balter for technical help.

## Conflict of interest

The authors declare that the research was conducted in the absence of any commercial or financial relationships that could be construed as a potential conflict of interest.

## Publisher’s note

All claims expressed in this article are solely those of the authors and do not necessarily represent those of their affiliated organizations, or those of the publisher, the editors and the reviewers. Any product that may be evaluated in this article, or claim that may be made by its manufacturer, is not guaranteed or endorsed by the publisher.

## References

[B1] Ahmad I. Rouf S. F. Sun L. Cimdins A. Shafeeq S. Le Guyon S. (2016). BcsZ inhibits biofilm phenotypes and promotes virulence by blocking cellulose production in *Salmonella enterica* serovar typhimurium. Microb. Cell Fact. 15 (1), 177. doi: 10.1186/s12934-016-0576-6 27756305 PMC5070118

[B2] Alegbeleye O. O. Singleton I. Sant’Ana A. S. (2018). Sources and contamination routes of microbial pathogens to fresh produce during field cultivation: A review. Food Microbiol. 73, 177–208. doi: 10.1016/j.fm.2018.01.003 29526204 PMC7127387

[B3] Amrutha B. Sundar K. Shetty P. H. (2017). Study on *E. coli* and *Salmonella* biofilms from fresh fruits and vegetables. J. Food Sci. Technol. 54 (5), 1091–1097. doi: 10.1007/s13197-017-2555-2 28416858 PMC5380633

[B4] Barak J. D. Gorski L. Naraghi-Arani P. Charkowski A. O. (2005). *Salmonella enterica* virulence genes are required for bacterial attachment to plant tissue. Appl. Environ. Microbiol. 71 (10), 5685–5691. doi: 10.1128/AEM.71.10.5685-5691.2005 16204476 PMC1265987

[B5] Barak J. D. Jahn C. E. Gibson D. L. Charkowski A. O. (2007). The role of cellulose and O-antigen capsule in the colonization of plants by *Salmonella enterica* . Mol. Plant Microbe Interact. 20 (9), 1083–1091. doi: 10.1094/MPMI-20-9-1083 17849711

[B6] Bayer E. A. Morag E. Wilchek M. Lamed R. Yaron S. Shoham Y. (1995). Cellulosome domains for novel biotechnological application. Prog. Biotechnol. 10, 251–259. doi: 10.1016/S0921-0423(06)80108-5

[B7] Bellincampi D. Cervone F. Lionetti V. (2014). Plant cell wall dynamics and wall-related susceptibility in plant-pathogen interactions. Front. Plant Sci. 5, 228. doi: 10.3389/fpls.2014.00228 24904623 PMC4036129

[B8] Ben-Barak Z. Streckel W. Yaron S. Cohen S. Prager R. Tschape H. (2006). The expression of the virulence-associated effector protein gene *avrA* is dependent on a *Salmonella enterica*-specific regulatory function. Int. J. Med. Microbiol. 296 (1), 25–38. doi: 10.1016/j.ijmm.2005.08.004 16377240

[B9] Berger C. N. Brown D. J. Shaw R. K. Minuzzi F. Feys B. Frankel G. (2011). *Salmonella enterica* strains belonging to O serogroup 1,3,19 induce chlorosis and wilting of *Arabidopsis thaliana* leaves. Environ. Microbiol. 13 (5), 1299–1308. doi: 10.1111/j.1462-2920.2011.02429.x 21349136

[B10] Brandl M. T. (2006). Fitness of human enteric pathogens on plants and implications for food safety. Annu. Rev. Phytopathol. 44, 367–392. doi: 10.1146/annurev.phyto.44.070505.143359 16704355

[B11] Carstens C. K. Salazar J. K. Darkoh C. (2019). Multistate outbreaks of foodborne illness in the United States associated with fresh produce from 2010 to 2017. Front. Microbiol. 10, 2667. doi: 10.3389/fmicb.2019.02667 31824454 PMC6883221

[B12] Chalupowicz L. Nissan G. Brandl M. T. McClelland M. Sessa G. Popov G.. (2018). Assessing the ability of *Salmonella enterica* to translocate type III effectors into plant cells. Mol. Plant Microbe Interact. 31 (2), 233–239. doi: 10.1094/MPMI-07-17-0166-R 28952399

[B13] Crook P. D. Aguilera J. F. Threlfall E. J. O’Brien S. J. Sigmundsdottir G. Wilson D.. (2003). A European outbreak of *Salmonella enterica* serotype typhimurium definitive phage type 204b in 2000. Clin. Microbiol. Infect. 9 (8), 839–845. doi: 10.1046/j.1469-0691.2003.00655.x 14616705

[B14] Datsenko K. A. Wanner B. L. (2000). One-step inactivation of chromosomal genes in *Escherichia coli* K-12 using PCR products. Proc. Natl. Acad. Sci. U.S.A. 97 (12), 6640–6645. doi: 10.1073/pnas.120163297 10829079 PMC18686

[B15] European Food Safety Authority (2017). “The European union summary report on trends and sources of zoonoses, zoonotic agents and food-borne outbreaks in 2016.” EFSA journal 2017; 15 (12) :5077. 10.2903/j.efsa.2017.5077 PMC7009962 32625371

[B16] Fisher N. Bourne A. Plunkett D. (2015). “Outbreak alert! 2015: A review of foodborne illness in the US from 2004-2013.” Center for Science in the Public Interest; 7th Edition, 2015

[B17] Gibson D. M. King B. C. Hayes M. L. Bergstrom G. C. (2011). Plant pathogens as a source of diverse enzymes for lignocellulose digestion. Curr. Opin. Microbiol. 14 (3), 264–270. doi: 10.1016/j.mib.2011.04.002 21536481

[B18] Gough N. M. Gearing D. P. King J. A. Willson T. A. Hilton D. J. Nicola N. A.. (1988). Molecular cloning and expression of the human homologue of the murine gene encoding myeloid leukemia-inhibitory factor. Proc. Natl. Acad. Sci. U.S.A. 85 (8), 2623–2627. doi: 10.1073/pnas.85.8.2623 3128791 PMC280050

[B19] Haft D. H. Selengut J. D. White O. (2003). The TIGRFAMs database of protein families. Nucleic Acids Res. 31 (1), 371–373. doi: 10.1093/nar/gkg128 12520025 PMC165575

[B20] Hartog E. Ben-Shalom L. Shachar D. Matthews K. R. Yaron S. (2008). Regulation of *marA*, *soxS*, *rob*, *acrAB* and *micF* in *Salmonella enterica* serovar typhimurium. Microbiol. Immunol. 52 (12), 565–574. doi: 10.1111/j.1348-0421.2008.00075.x 19120970

[B21] Hematy K. Cherk C. Somerville S. (2009). Host-pathogen warfare at the plant cell wall. Curr. Opin. Plant Biol. 12 (4), 406–413. doi: 10.1016/j.pbi.2009.06.007 19616468

[B22] Jeter C. Matthysse A. G. (2005). Characterization of the binding of diarrheagenic strains of *E. coli* to plant surfaces and the role of curli in the interaction of the bacteria with alfalfa sprouts. Mol. Plant Microbe Interact. 18 (11), 1235–1242. doi: 10.1094/MPMI-18-1235 16353558

[B23] Jha G. Rajeshwari R. Sonti R. V. (2007). Functional interplay between two *Xanthomonas oryzae* pv. *oryzae* secretion systems in modulating virulence on rice. Mol. Plant Microbe Interact. 20 (1), 31–40. doi: 10.1094/MPMI-20-0031 17249420

[B24] Kisluk G. Kalily E. Yaron S. (2013). Resistance to essential oils affects survival of *Salmonella enterica* serovars in growing and harvested basil. Environ. Microbiol. 15 (10), 2787–2798. doi: 10.1111/1462-2920.12139 23648052

[B25] Kisluk G. Yaron S. (2012). Presence and persistence of *Salmonella enterica* serotype typhimurium in the phyllosphere and rhizosphere of spray-irrigated parsley. Appl. Environ. Microbiol. 78 (11), 4030–4036. doi: 10.1128/AEM.00087-12 22447598 PMC3346414

[B26] Kongruang S. Han M. J. Breton C. I. Penner M. H. (2004). Quantitative analysis of cellulose-reducing ends. Appl. Biochem. Biotechnol. 113-116, 213–231. doi: 10.1385/ABAB:113:1-3:213 15054208

[B27] Kroupitski Y. Pinto R. Belausov E. Sela S. (2011). Distribution of *Salmonella* typhimurium in romaine lettuce leaves. Food Microbiol. 28 (5), 990–997. doi: 10.1016/j.fm.2011.01.007 21569943

[B28] Kroupitski Y. Pinto R. Brandl M. T. Belausov E. Sela S. (2009). Interactions of *Salmonella enterica* with lettuce leaves. J. Appl. Microbiol. 106 (6), 1876–1885. doi: 10.1111/j.1365-2672.2009.04152.x 19239550

[B29] Kubicek C. P. Starr T. L. Glass N. L. (2014). Plant cell wall-degrading enzymes and their secretion in plant-pathogenic fungi. Annu. Rev. Phytopathol. 52, 427–451. doi: 10.1146/annurev-phyto-102313-045831 25001456

[B30] Lapidot A. Romling U. Yaron S. (2006). Biofilm formation and the survival of *Salmonella* typhimurium on parsley. Int. J. Food Microbiol. 109 (3), 229–233. doi: 10.1016/j.ijfoodmicro.2006.01.012 16616389

[B31] Lapidot A. Yaron S. (2009). Transfer of *Salmonella enterica* serovar typhimurium from contaminated irrigation water to parsley is dependent on curli and cellulose, the biofilm matrix components. J. Food Prot. 72 (3), 618–623. doi: 10.4315/0362-028X-72.3.618 19343953

[B32] Lehmann G. Ziv T. Braten O. Admon A. Udasin R. G. Ciechanover A. (2016). Ubiquitination of specific mitochondrial matrix proteins. Biochem. Biophys. Res. Commun. 475 (1), 8–13. doi: 10.1016/j.bbrc.2016.04.150 27157140

[B33] Martinez-Vaz B. M. Fink R. C. Diez-Gonzalez F. Sadowsky M. J. (2014). Enteric pathogen-plant interactions: Molecular connections leading to colonization and growth and implications for food safety. Microbes Environ. 29 (2), 123–135. doi: 10.1264/jsme2.ME13139 24859308 PMC4103518

[B34] Matthysse A. G. Thomas D. L. White A. R. (1995). Mechanism of cellulose synthesis in *Agrobacterium tumefaciens* . J. Bacteriol. 177 (4), 1076–1081. doi: 10.1128/jb.177.4.1076-1081.1995 7860586 PMC176704

[B35] Mazur O. Zimmer J. (2011). Apo- and cellopentaose-bound structures of the bacterial cellulose synthase subunit BcsZ. J. Biol. Chem. 286 (20), 17601–17606. doi: 10.1074/jbc.M111.227660 21454578 PMC3093835

[B36] Montano J. Rossidivito G. Torreano J. Porwollik S. Sela Saldinger S. McClelland M.. (2020). *Salmonella enterica* serovar typhimurium 14028s genomic regions required for colonization of lettuce leaves. Front. Microbiol. 11: 6. doi: 10.3389/fmicb.2020.00006 PMC6993584 32038592

[B37] Roberts D. P. Denny T. P. Schell M. A. (1988). Cloning of the *egl* gene *of pseudomonas solanacearum* and analysis of its role in phytopathogenicity. J. Bacteriol. 170 (4), 1445–1451. doi: 10.1128/jb.170.4.1445-1451.1988 2832363 PMC210987

[B38] Robledo M. Rivera L. Jimenez-Zurdo J. I. Rivas R. Dazzo F. Velazquez E.. (2012). Role of *Rhizobium* endoglucanase CelC2 in cellulose biosynthesis and biofilm formation on plant roots and abiotic surfaces. Microb. Cell Fact. 11, 125. doi: 10.1186/1475-2859-11-125 22970813 PMC3520766

[B39] Romling U. Galperin M. Y. (2015). Bacterial cellulose biosynthesis: diversity of operons, subunits, products, and functions. Trends Microbiol. 23 (9), 545–557. doi: 10.1016/j.tim.2015.05.005 26077867 PMC4676712

[B40] Schikora A. Carreri A. Charpentier E. Hirt H. (2008). The dark side of the salad: *Salmonella* typhimurium overcomes the innate immune response of *Arabidopsis thaliana* and shows an endopathogenic lifestyle. PloS One 3 (5), e2279. doi: 10.1371/journal.pone.0002279 18509467 PMC2386236

[B41] Shaw R. K. Lasa I. Garcia B. M. Pallen M. J. Hinton J. C. Berger C. N.. (2011). Cellulose mediates attachment of *Salmonella enterica* serovar typhimurium to tomatoes. Environ. Microbiol. Rep. 3 (5), 569–573. doi: 10.1111/j.1758-2229.2011.00263.x 23761337

[B42] Shirron N. Kisluk G. Zelikovich Y. Eivin I. Shimoni E. Yaron S. (2009). A comparative study assaying commonly used sanitizers for antimicrobial activity against indicator bacteria and a Salmonella Typhimurium strain on fresh produce. J Food Prot 72 (11):2413-2417. doi: 10.4315/0362-028x-72.11.2413 19903410

[B43] Shirron N. Yaron S. (2011). Active suppression of early immune response in tobacco by the human pathogen *Salmonella* typhimurium. PloS One 6 (4), e18855. doi: 10.1371/journal.pone.0018855 21541320 PMC3082535

[B44] Solano C. Garcia B. Valle J. Berasain C. Ghigo J. M. Gamazo C.. (2002). Genetic analysis of *Salmonella enteritidis* biofilm formation: critical role of cellulose. Mol. Microbiol. 43 (3), 793–808. doi: 10.1046/j.1365-2958.2002.02802.x 11929533

[B45] Solnik- Isaac H. Tabak M. Ben-David A. Shachar D. Weinberger M. Yaron S. (2007). Quinolone resistance of *Salmonella enterica* serovar virchow isolates from humans and poultry in Israel: evidence for clonal expansion. J. Clin. Microbial. 45, 2575–2579. doi: 10.1128/JCM.00062-07 PMC1951243 17596371

[B46] Souza C. A. Li S. Lin A. Z. Boutrot F. Grossmann G. Zipfel C.. (2017). Cellulose-derived oligomers act as damage-associated molecular patterns and trigger defense-like responses. Plant Physiol. 173 (4), 2383–2398. doi: 10.1104/pp.16.01680 28242654 PMC5373054

[B47] Szpinak V. Ganz M. Yaron S. (2022). Factors affecting the thermal resistance of *Salmonella* typhimurium in tahini. Food Res. Int. 155, 111088. doi: 10.1016/j.foodres.2022.111088 35400463

[B48] Tan M. S. Moore S. C. Tabor R. F. Fegan N. Rahman S. Dykes G. A. (2016). Attachment of *Salmonella* strains to a plant cell wall model is modulated by surface characteristics and not by specific carbohydrate interactions. BMC Microbiol. 16, 212. doi: 10.1186/s12866-016-0832-2 27629769 PMC5024418

[B49] Teather R. M. Wood P. J. (1982). Use of Congo red-polysaccharide interactions in enumeration and characterization of cellulolytic bacteria from the bovine rumen. Appl. Environ. Microbiol. 43 (4), 777–780. doi: 10.1128/aem.43.4.777-780.1982 7081984 PMC241917

[B50] Teplitski M. Noel J. T. Alagely A. Danyluk M. D. (2012). Functional genomics studies shed light on the nutrition and gene expression of non-typhoidal *Salmonella* and enterovirulent *E. coli* in produce. Food Res. Int. 45, 576–586. doi: 10.1016/j.foodres.2011.06.020

[B51] Vestby L. K. Moretro T. Langsrud S. Heir E. Nesse L. L. (2009). Biofilm forming abilities of *Salmonella* are correlated with persistence in fish meal- and feed factories. BMC Vet. Res. 5, 20. doi: 10.1186/1746-6148-5-20 19473515 PMC2693496

[B52] Walton J. D. (1994). Deconstructing the cell wall. Plant Physiol. 104 (4), 1113–1118. doi: 10.1104/pp.104.4.1113 12232152 PMC159271

[B53] Wang X. Dubey A. K. Suzuki K. Baker C. S. Babitzke P. Romeo T. (2005). CsrA post-transcriptionally represses *pgaABCD*, responsible for synthesis of a biofilm polysaccharide adhesin of *Escherichia coli* . Mol. Microbiol. 56 (6), 1648–1663. doi: 10.1111/j.1365-2958.2005.04648.x 15916613

[B54] Weinberger M. Yaron S. Agmon V. Yishai R. Rosenberg A. Peretz C. (2011). Curtailed short-term and long-term survival following infection with non-typhoid *Salmonella* in israel. Clin. Microbiol. Infect. 17 (2), 278–284. doi: 10.1111/j.1469-0691.2010.03184.x 20132249

[B55] Xia T. Li Y. Sun D. Zhuo T. Fan X. Zou H. (2016). Identification of an extracellular endoglucanase that is required for full virulence in *Xanthomonas citri* subsp. *citri* . PloS One 11 (3), e0151017. doi: 10.1371/journal.pone.0151017 26950296 PMC4780785

[B56] Yaron S. Romling U. (2014). Biofilm formation by enteric pathogens and its role in plant colonization and persistence. Microb. Biotechnol. 7 (6), 496–516. doi: 10.1111/1751-7915.12186 25351039 PMC4265070

[B57] Yoo J. S. Jung Y. J. Chung S. Y. Lee Y. C. Choi Y. L. (2004). Molecular cloning and characterization of CMCase gene (*celC*) from *Salmonella* typhimurium UR. J. Microbiol. 42 (3), 205–210. 15459649

[B58] Zaidenstein R. Peretz C. Nissan I. Reisfeld A. Yaron S. Agmon V.. (2010). The epidemiology of extraintestinal non-typhoid *Salmonella* in Israel: the effects of patients’ age and sex. Eur. J. Clin. Microbiol. Infect. Dis. 29 (9), 1103–1109. doi: 10.1007/s10096-010-0968-1 20535625

[B59] Zhang Y. H. Lynd L. R. (2005). Determination of the number-average degree of polymerization of cellodextrins and cellulose with application to enzymatic hydrolysis. Biomacromolecules 6 (3), 1510–1515. doi: 10.1021/bm049235j 15877372

