# Supplementary material for: The activity of BcsZ of Salmonella Typhimurium and its role in Salmonella-plants interactions
Source: Front Cell Infect Microbiol. 2022 Aug 23;12:967796. doi: 10.3389/fcimb.2022.967796 (PMC9445439; doi:10.3389/fcimb.2022.967796)
Supplement: Supplementary file 1 [file DataSheet_1.docx]

Supplementary Material

# Supplementary Figures and Tables

##
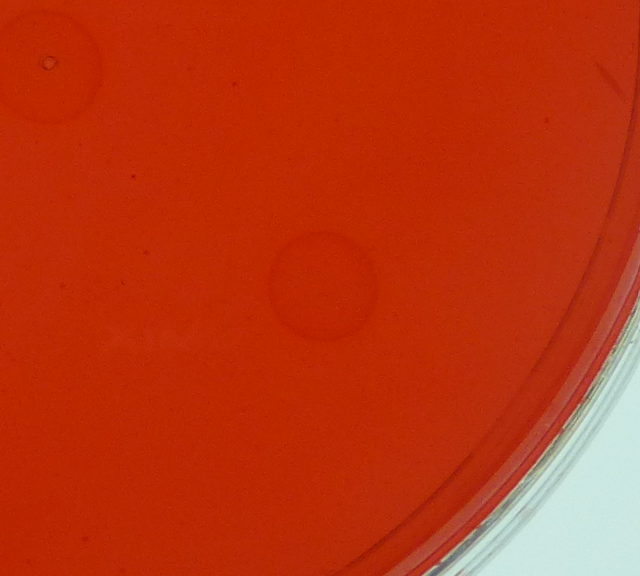

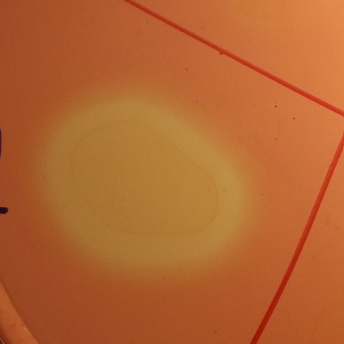
Supplementary Figure S1.

A.

B.

**Supplementary Figure S1.** Degradation of carboxymethylcellulose (CMC) on CMC agar plates by *S*. Typhimurium (w.t.) intact cells (A) and ΔBcsZ harboring a pBcsZ plasmid (B). The plates were stained with 0.1% congo red and washed with 1M NaCl. Bright halos indicate CMC degradation.

**
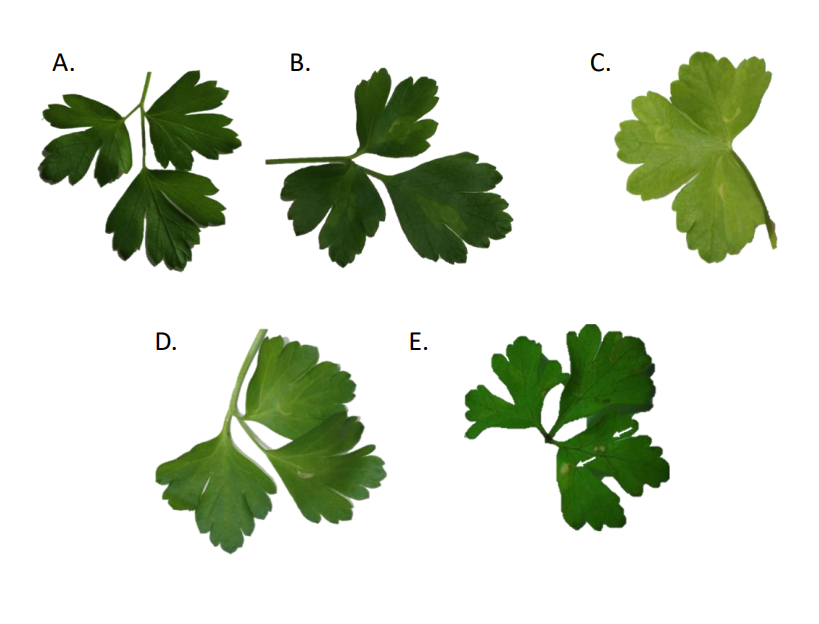
**

**Supplementary Figure S2**. **Damage gradient following infiltration**. Leaves that were infiltrated with different bacterial strains were scored by their leaf damage from 1- no necrotic damage to 5 the highest leaf damage. **A**. Score-1, no damage to leaf. **B**. Score-2, infiltration is marked but there is no change in leaf color. **C**. Score-3, the color of the infiltrated spot changed from green to yellow-brown. **D**. Score-4, the color of the infiltrated spot changed to brown and small cavities appeared. **E**. Score-5, the infiltrated spots bleached and have cavities not only in the infiltrated spots but also in other locations on the leaf.

## Supplementary Figure S3


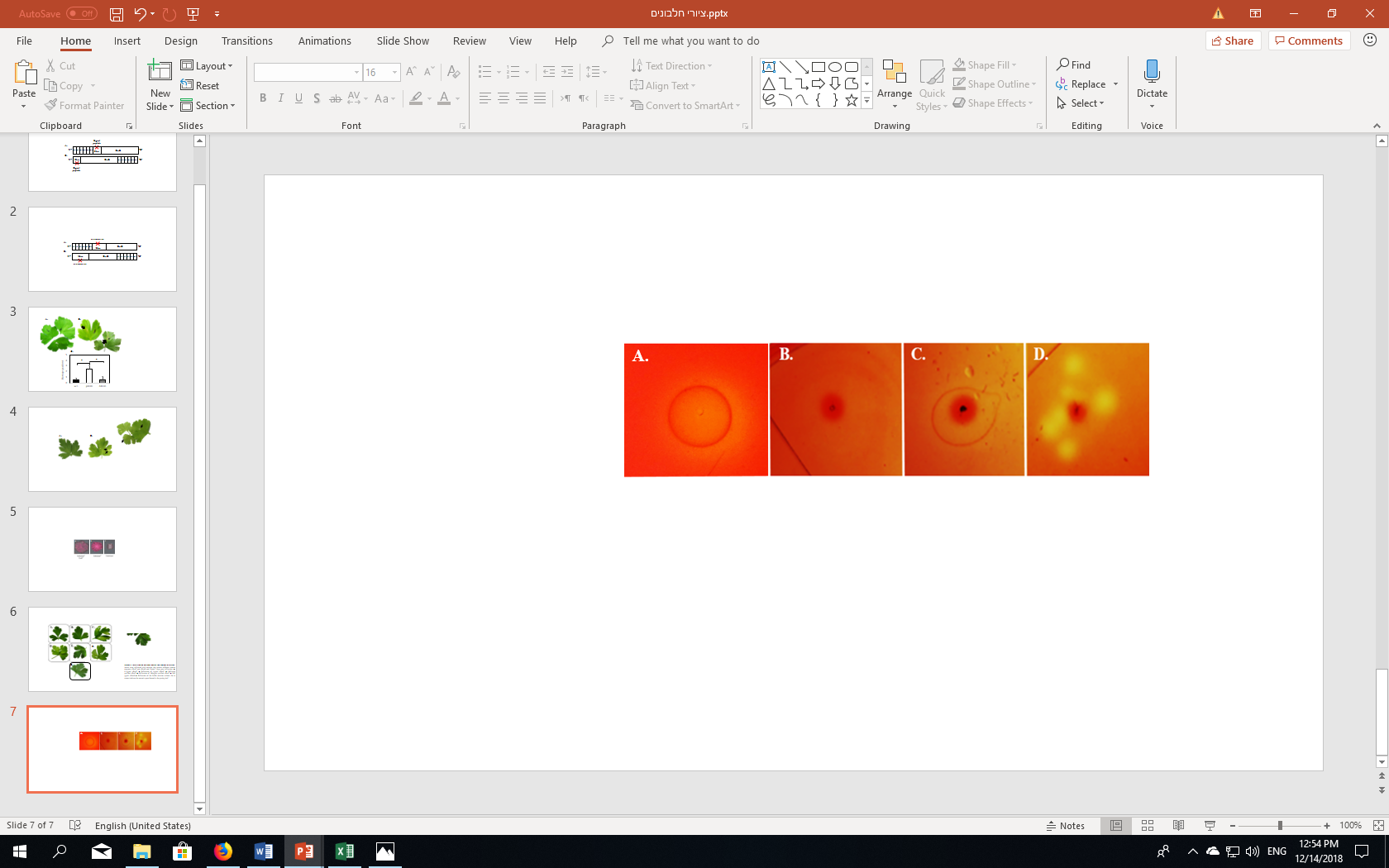


**Supplementary Figure S3**. Degradation of CMC agar plates by different *Salmonella* strains. Each *Salmonella* lysate was plated on 1% CMC agar plate and stained with congo red to examine CMC degradation ability **A**. *S*. Typhimurium ATCC14028 strain **B**. *S*. Typhimurium 8300 strain **C**. *S*. Typhimurium 153-1 strain **D**. *S*. Agona strain 71-4.

## Supplementary Table S1

| **Gene name** | **Sequence (5'-3') [nmol/L]** | - **Application** | - **Ta (°C)** |
| --- | --- | --- | --- |
| *bcsZ* | Fw: AAACCATGGCATGTAGCTGGCCTGCGTGG   - Rev:GTATTGGGATCCTTAGTGGTGGTGGTGTGGTGCGTGAACTTACGCATTCCTG | Cloning to  pET-9d | 60 |
| *bcsZ* | Fw:AGGGAATTCATGATGACTATGCTGCGCGGATGG   - Rev:AGGTCTAGATTAGTGTGAATTTGCGCATTC | Cloning to pCA114 | 58 |
| *bcsZ* | - Fw: CCGTCAAAGGAACCGTAAA - Rev: TGCCCGGTAAACAGATGAG | Site directed mutagenesis of  *bcsZ* | - 58 |
| *∆bcsZ* | - Fw: ATTACTGGCGTGGGTATTGTGGCGTCTGCTACGT - Rev: TTGGCGAAAATGAGACGTTGATCGGCACGTA | Knockout verification | - 60 |
| pKD46 | - Fw: GGGAATTCGAGCTCTAAGGAGG - Rev: GGTGATCAGTTCCTGTGGGT | - Identification of pKD46 plasmid | - 58 |
| - pKD3-cat | - Fw: GTGTAGGCTGGAGCTGCTTC - Rev: ATGGGAATTAGCCATGGTCC | - Identification of Cm^R^ cassette | - 58 |
| - *bcsABZC* | - CTGGGATCCAAGCGGAAACACTATCCACGC | - Promoter site | - 58 |
| - *bcsEFG* | - CTGGGATCCTTTGTGACGCAATCGTTTGATT | - Promoter site | - 58 |

**
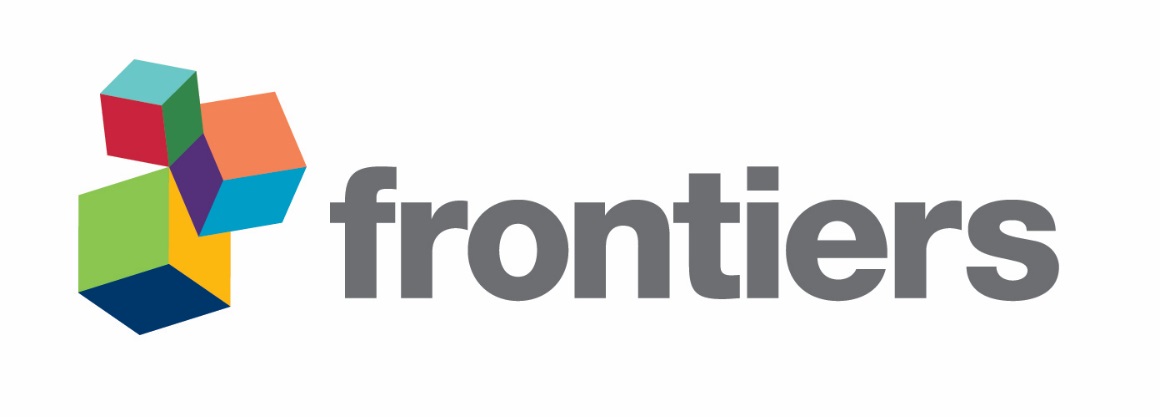
**
